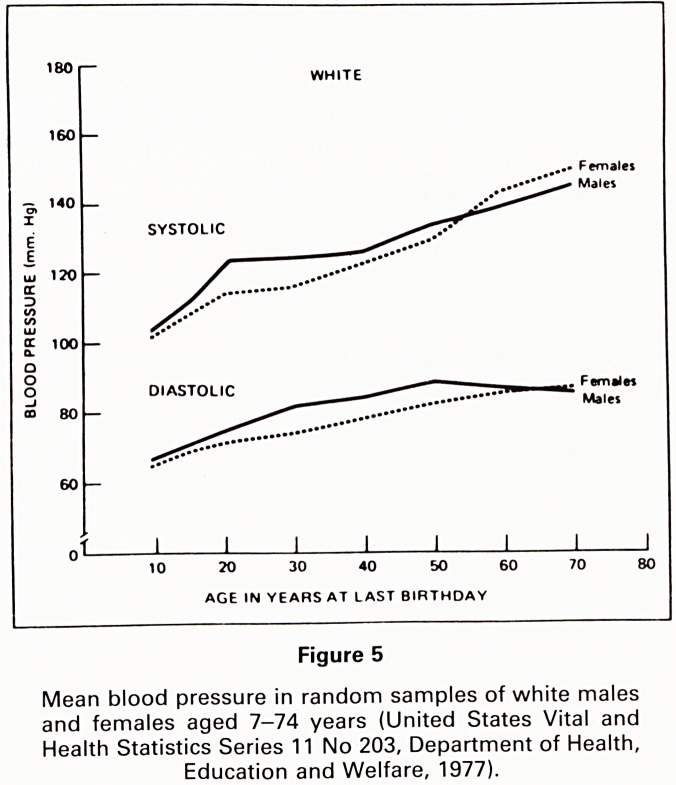# Appreciating Hypertension, Quicksands and Firm Ground

**Published:** 1986-10

**Authors:** D. W. Barritt

**Affiliations:** Emeritus Consultant Physician, Bristol Royal Infirmary


### Appreciating Hypertension, Quicksands and Firm Ground

**D. W. Barritt, MD, FRCP**
Emeritus Consultant Physician, Bristol Royal Infirmary

**Based on the Carey Coombs Memorial Lecture 1986 to the University of Bristol**

Carey Franklin Coombs achieved great distinction as a physician and pioneer of cardiac research despite a relatively early death at the age of 52 years. At Bristol General Hospital he organised a University Centre of Cardiac Research where he pursued his studies of rheumatic heart disease, cardiovascular syphilis and the aetiology of other cardiac diseases, Lawrence O'Shaughnessy gave the first Carey Coombs Memorial Lecture in 1937 on the subject of cardio omentopexy in the treatment of angina pectoris. Of the dozen or so Carey Coombs lectures since the war, only one has dealt with the subject of hypertension, that given by Sir Stanley Peart on renal hypertension in 1972.

Fifteen years ago, medication for hypertension was complex not to say chaotic. One personal landmark was the referral of a patient who was discharged from a specialist hypertension unit taking no less than 40 tablets a day. It seemed a good field for study.

The first problem with hypertension is to decide what it is and perhaps it would be simpler if we stopped regarding hypertension as a disease. After all, rises in blood pressure produce no discomfort. Those whose daily lives are accompanied by systolic pressures close to 200 mmHg are at no disadvantage compared to those with only half that pressure. The fact that stroke, coronary disease and heart failure may be accelerated by high arterial pressures has a close parallel with the effect of cigarette smoking. Is that a disease? Is the excess consumption of animal fat a disease, are the overeating of the obese, gross physical sloth or excess consumption of alcohol diseases? With high blood pressure there is the added difficulty of its fluctuation from minute to minute. It has been well known since pressures could first be measured that normal blood pressure varies from minute to minute. Ability to record blood pressure continuously from beat to beat through an indwelling arterial needle has highlighted and clarified the extent of this variation. Fig. 1 shows the circadian nature of blood pressure fluctuation through the day and that this waking and sleeping pattern is maintained also in hypertension. The circadian BP pattern is accompanied by parallel changes in heart rate which suggest that the sympathetic nervous system is responsible. These are mean figures from a group. Records in individuals also show the high levels that normals will exhibit during moments of stress and exertion so that at the peak of sexual orgasm for instance, systolic pressures may rise as high as 300 mmHg in all of us.

With this background the occasional measurement of blood pressure by the sphygmomanometer on hospital or surgery premises seems a hopelessly poor way of deciding if a blood pressure figure is to be regarded as normal or abnormal. And if one reading of blood pressure were to be the only basis on which to decide whom to treat this would be entirely true. Fortunately short term variations of blood pressure need not be entirely misleading.

In the first place, in population studies a single reading of blood pressure, or perhaps more than one reading at one examination has been used to assess the long term risks of higher levels of blood pressure. The results are remarkably consistent in showing that in statistical terms the risk of damage to health and the risk of death rises progressively from one group of people to the next who have higher and higher levels of pressure. Thus in the study of Whitehall Civil Servants following up 18,000 men for five years, the 20% with pressures above 152/95 mmHg were 2½ times as likely to die of coronary heart disease as those with the lowest pressures. Fig. 2. Looking at deaths from all causes the same message

**Figure 2**

5-year mortality rates from coronary heart disease according to initial blood pressure among men in the Whitehall Study.

**Figure 3**

Association between 'casual' blood pressure and mortality, expressed as age-specific relative risk (Build and Blood Pressure Study of US Society of Actuaries).

**Figure 4**

Differences between smokers and non-smokers of cigarettes in the mortality from coronary heart disease at various levels of blood pressures. (Data from the Whitehall Study).

**Figure 5**

Mean blood pressure in random samples of white males and females aged 7–74 years (United States Vital and Health Statistics Series 11 No 203, Department of Health, Education and Welfare, 1977).

| | |
|---|---|
| AFRICA | CONGO PYGMIES<br>KALAHARI BUSHMEN<br>KENYAN NOMADS |
| AMERICA | CUNA INDIANS, PANAMA |
| ASIA | NEW GUINEA HIGHLANDERS<br>PACIFIC ATOLL POLYNESIANS |
| AUSTRALIA | ABORIGINES |

emerges that for each age group, relative mortality is three times higher in those with systolic pressures between 160 and 165 than in those whose systolic pressure is less than 130 mmHg Fig. 3. We should not forget, of course, that other factors may be equally powerful so that in the same study the addition of cigarette smoking to blood pressure increase, at least doubled the risk of death from coronary disease. Fig. 4.

The fact that population studies of casual blood pressure readings are meaningful and repay study enables us to look with some profit at the geographical and racial distribution of hypertension and consider what causes blood pressure to rise.

In all parts of the world there will be a low incidence of hypertension and its complications due to chronic renal disease, endocrine tumours, and aortic coarctation. In nearly all parts of the world disadvantageously high blood pressure in around 10% of the population is present without a discoverable cause. In these populations mean blood pressure tends to rise slowly with age. Fig. 5. In just a few parts of the world, out of reach of the television, canned food and motor cars no such rise with age is found and primary hypertension is absent. Table I shows how small is the portion of the world still so pure.

What is also clear, as is the case with coronary disease, is that this absence of the trigger for blood pressure rise is not due to differences of race but to life style, for those members of the unaffected races who migrate to mechanised cultures lose their immunity to primary hypertension. Again as is the case with coronary disease the background mechanisms are complex and remain in doubt.

Although the absence of primary hypertension cannot be due to genetic differences between races it is clear that genetic influence is strong as a determinant of blood pressure levels. Indeed so strong is it that it was possible for the last generation of medical science's grand old men to argue with passion that the cause might be in a single gene. The argument proved to be a quicksand and the truth emerged that many factors must operate to control levels of blood pressure, strong as is the incidence of hypertension in many families. Studies of monozygotic and dizygotic twins and of adopted children confirm the importance of the genetic factor but clearly it will only operate in the correct environmental setting.

We seem to be on firm ground therefore in blaming the unduly common rise of blood pressure with age on to the mode of life in mechanised societies. When we try to isolate the trigger factors it is quicksands all the way. Almost nothing in present day European life equates to that of primitive society except for the universal passions to compete, to quarrel and to make love.

Most suspicion has fallen on the artificially high dietary intake of salt and on the undefinable factor of psychological stress. As salt in the diet and salt in the body fluids can be weighed and measured there is a considerable literature devoted to the subject of its importance in the pathogenesis of hypertension. No clear answer emerges as yet. This is not too surprising when one considers as a starting point that normal man may tolerate a sodium intake as low as 30 milli-equivalents per day or as high as 150 without any apparent ill effect or any influence on blood pressure. The two populations that have been cited as evidence for the theory that dietary salt is crucial are those primitive people cited in table I, whose daily salt intake is of the order of 30 milli-equivalents per day or less and whose blood pressure stays low up to the age of 60. On the other hand there are the Northern Japanese whose daily intake may be above 300 milli-equivalents per day and among whom severe hypertension is unduly common.

In societies such as ours numerous studies have failed to find any association between the daily salt intake and the height of the blood pressure. It may well be of course that given a sufficient daily salt intake a genetic factor or other additional factors are necessary in combination to cause hypertension. A common sense conclusion in the absence of firm ground would seem to be that with no certainty as to its importance we cannot recommend troublesome culinary effort to rid the diet of salt but the addition of packeted salt in quantity in the preparation of food is quite unnecessary and possibly harmful. Difficult as is the evaluation of salt intake in the causation of hypertension the importance of other dietary factors presents even greater problems of assessment. That they may be important receives support from a recent study from Western Australia that vegetarians have lower blood pressure than meat eaters and are less likely to develop hypertension. The effect is greater than would be accounted for by the absence of obesity and the lower salt intake of vegetarians. The important association between obesity and hypertension also underlines the role of dietary factors. In a recent report from Milwaukee two-thirds of those patients presenting to a hypertension clinic were overweight or frankly obese and only a handful underweight. Obesity, however, seemed not to be associated with severe hypertension, in most subjects blood pressure rise was modest.

High levels of alcohol consumption seem to be yet another dietary factor causing the blood pressure to rise. Cigarette smoking does not although of course it greatly increases the risk of death and disability in hypertensives.

As with coronary disease there seems to be an irresistable urge to believe that mental stress is a major causative factor in hypertension. The starting point seems to be the doubtful assumption that stress is virtually absent in primitive societies who pluck their every meal from the soil or the sea and fight their neighbours with spears and arrows and are worried by evil spirits, and yet is ever more pressing in communities where food and shelter are relatively abundant and where life expectancy is altogether more secure. It is easy to prove that mental effort and fright cause an instantaneous rise of blood pressure in all subjects but impossible as yet to prove that this is a mechanism capable of producing a lifelong rise of pressure.

The possible causative mechanisms considered so far apply to more than 90% of hypertensive people. However, half a century of intensive and world wide laboratory and clinical research has clarified some patho-physiological mechanisms which will produce severe hypertension and at times enthusiasts have believed that such identifiable disturbances may be present in the majority of hypertensives. Disease of one or both kidneys is the biggest group, endocrine based disorders the next and hypertension related to long term use of the contraceptive pill of some numerical importance.

Every doctor who discovers a rise in blood pressure in one of his patients has to decide how many investigations to perform to exclude such conditions. John Ledingham opens his chapter in the Bristol based book 'The Hypertensive Patient' with the sentences 'a great deal of effort is made by physicians to find an underlying cause when patients present to them with raised arterial pressure. The frequency with which they find such a cause depends on the population of patients with whom they deal, and the length to which they take investigation...'. Past experience shows that if every hypertensive patient is submitted to an intravenous pyelogram, an isotope renal scan and bilateral renal arteriograms minor abnormalities will quite commonly be found. They are usually irrelevant to the management of the patient. Clearly the detection of renal disease may be crucial to prognosis and the removal of an adrenal tumour may save years of tablet taking but my own practice was not to ask for radiological examinations or extensive laboratory tests if a careful history and clinical examination raised no suspicions and if the urine was free of albumin and if serum sodium, potassium and urea concentrations were normal. Failure of high pressure to fall with well directed medication, however, calls for very thorough reappraisal. Concomitant with the decision as to the extent of laboratory investigation is the decision as to whether or not treatment is urgent. At the first consultation only the presence of excessively high pressure levels or retinal haemorrhage with or without papilloedema calls for immediate therapy.

Retinal haemorrhage implies persistently high pressure with the risk of cerebral haemorrhage and vascular damage to the kidney both of which are virtually removed by pressure lowering therapy. There is no dispute that patients with severe hypertension benefit from the prescription of drugs which lower pressure. Placebo-controlled trials were not needed to prove the benefit of medication in those patients with papilloedema or retinal haemorrhage and albuminuria which signal the malignant phase, for it was long known that in these circumstances almost all patients are dead within two years. With therapy the prognosis is transformed with relative ease. For the higher grades of pressure elevation without papilloedema placebo-controlled trials were necessary but all have shown the benefit of treatment in terms of a reduction in mortality and the incidence of disabling strokes.

For the lesser degrees of pressure elevation fairly massive placebo-controlled trials in several countries have produced results which make the decision as to whether to medicate still debatable for each individual patient.

Before considering the rather baffling statistics of these latter trials we should appreciate the problems of current therapy of those who unquestionably need it. A few years ago it was emminently sensible to say that the practising physician need worry less about the problems of mild hypertension than the clear necessity to treat those with severe hypertension well. It is still the case that far too many patients with severe hypertension are unknown, untreated or rather poorly treated.

Lest we be accused of neglecting the whole person for a blinkered prescription of powerful drugs it needs to be said that general measures should always be discussed. First consideration must always be 'do you smoke?' for the risk of vascular complications with their associated risk to life are at least doubled if not tripled in smokers.

Cigarette smoking has to be banned with all the severity we can muster. We ought to talk about diet. For the overweight dietary restriction brings the real benefits of a reduction in the risk of maturity onset diabetes, relief for weight bearing joints a real social asset and in most cases a small fall in intra-arterial pressure in addition to the sphygmomanometer reading which is falsely elevated by a fat arm. Sensible eating habits bring benefits to all and these may well include some lessening of the risk of coronary disease from which the hypertensive patient has most to fear. Next our patients will expect to be given guidance about everyday activities and will perhaps assume that they are going to be told (or even say they have been told when they have not) that they must ease up. There are no scientific rules to rely on here. Blood pressure falls with rest, relaxation and sleep and rises with physical, psychological and emotional stress. If you tell your patient to avoid stressful situations at work are you also going to tell him or her not to put too much into family life and leisure pursuits. Let those who like going to gurus go, but with the expectation that it will not relieve us of the difficulty in deciding whether or not to prescribe a nuisance drug. When we do decide that medication is either essential or desirable individual preferences and prejudices of the doctor operate quite powerfully.

For some years beta blockade and diuretics have run neck and neck in popularity as first line drugs. This is because they are roughly equipotent, neither causes postural hypotension, or very commonly any other troublesome side effects and both can be taken once daily. An intriguing and rather annoying point is that it is still quite unclear as to how either drug lowers blood pressure. Neither was introduced into clinical practice with that intention. The pressure lowering action of diuretics depends on their natiuretic action and is negated by sodium replacement. Beta blockade depresses cardiac output, renin production and catecholamine levels but none of these three pharmacological actions of the drugs seem critical to its hypotensive action.

The main disadvantage of beta blockade are that it commonly causes uncomfortable coldness of the extremities and although it was not possible for us to be sure of any other unwanted side effects on small numbers of patients, the MRC trial involving thousands of patients gives figures of 12% for lassitude and 13% for impotence compared with smaller numbers for placebo tablets. There must presumably be some depression of peak physical exertion as cardiac output is depressed.

It was not difficult to establish that diuretics could induce clinical gout, diabetes and sometimes worrying hypokalaemia but again it took the size of the MRC trial to spot its ability to cause impotence in one or more men in ten. A restriction of dietary salt will usually cause some fall in blood pressure in hypertensive patients. That is with the use of such measures as not adding salt to food at the table and taking no tinned vegetables but using ordinary bread and ordinary butter. It is obviously nonsensical to prescribe diuretics without stating the need not to add salt to a diet.

Of course it is an important advantage of these two groups of drugs that their effect is independent and additive so that the two combined together double the hypotensive potency with little increase in side effects and requiring only one tablet daily. Of the other agents methyl dopa has been most widely used and its long term safety well established. The usually recommended doses frequently cause troublesome lassitude but if given once in the evening amounts as small as 250 or 500 mg. will give worthwhile additional pressure falls without side effects. Prazosin and hydrallazine rank fairly equally and the more recent introduction of enalapril and nifedipine may call for a revision of ranking order.

A clear understanding of the problems of drug therapy is the basis on which to consider the pros and cons of treating individual patients with less severe degrees of hypertension, whose risk of developing worsening renal failure, heart failure or cerebral haemorrhage is very substantially less. Quite clearly only large scale placebo-controlled trials can give any indication of what we can tell patients who are entirely symptomless. Such a trial was completed in Australia five years ago. Three and a half thousand patients were randomised to hypotensive therapy or matching placebo tablets over a four year period. There was a significant benefit of treatment in terms of deaths and non fatal stroke but the differences were relatively small and the number of tablets prescribed to save one life for a few years was very large. Thus of 170 patients taking a hypotensive tablet daily for four years only one would be saved from premature death and another one from stroke in that four year period. Results of the very large United Kingdom MRC trial published last year showed even less benefit from therapy. Over 17,000 patients were followed for five and a half years. Treatment seemed to save no lives. The main conclusion was: 'The trial has shown that if 850 mildly hypertensive patients are given active hypotensive drugs for one year about one stroke will be prevented. This is an important but infrequent benefit. Its achievement subjected a substantial percentage of the patients to chronic side effects, mostly but not all minor'.

As clinicians we should expect our studies to concentrate on the answer to the question 'How should we manage patients with high blood pressure'. Some recommendations can be regarded as beyond doubt, others as best bets for today.

The first is now beyond doubt and that is that every adult should have the blood pressure measured and recorded at suitable intervals. As hypertension of all grades of severity is usually symptomless screening for hypertension by general practitioners is or should be standard practice. If blood pressure is raised the two questions to be answered are, how much investigation is required and should medication be given. An important starting point is that except in the presence of papilloedema there is rarely any hurry to make decisions and it will almost always be correct just to retake the blood pressure at intervals of a week or a month or a year. An important proportion will be found to have pressure settling into the normal range with repeated measurements. In the MRC trial the patients entered had more than one reading of blood pressure on three separate occasions and yet 18% of those not treated had diastolic pressures below 90 mmHg at each of three annual follow up visits. Thus the first sensible step is to observe over a period of time which may be months or years how consistently the high blood pressure is. During this observation period the benefits of stopping smoking, reducing dietary salt intake and correcting obesity and limiting alcohol intake can be stressed. Both in terms of the individual patient and the fate of the whole group with mild hypertension much more will be gained by a few giving up the cigarette habit than by all being medicated. Ramsey has recently given his view that of all those with mild hypertension followed in this way only about 12% will require long term drug therapy.

Three things are mandatory. First to perform a clinical examination, palpate the abdomen for enlarged kidneys and to feel the femoral pulse. Second to examine the urine for protein and third to measure blood urea and electrolytes. The latter should detect those with important renal impairment or with Conn's syndrome.

*(continued on page 118)*

### Appreciating Hypertension *(continued from page 104)*

At this stage the approach to medication should be on a trial basis with two questions to be answered. Firstly is the treatment acceptable in that it causes no side effects and second does it lower the pressure? Unsatisfactory answers to both questions will lead to a change of therapy or an addition. If pressures fall consistently into or near the normal range it is quite appropriate to discontinue treatment and monitor the response.

Long term management can perfectly well be in the hands of well trained paramedical staff but decisions on initiating therapy and laying down clinical guidelines still call for high levels of knowledge and experienced judgement from every doctor who takes charge of any single patient with raised blood pressure.

**REFERENCES**

LEDINGHAM J. G. G. (1980) *In:* The Hypertensive Patient. p 212. The Pitman Press, Bath.

MacMAHON S. W., BLACKET R. B., MACDONALD G. J. and HALL W. (1984) Obesity, alcohol consumption and blood pressure in Australian men and women. *J. Hypertension,* **2**, 85–91.

Management Committee (1980) The Australian Therapeutic trial in mild hypertension. *Lancet* **1**, 1261.

Medical Research Council Working Party (1981) Adverse reactions to bendrofluazide and propranolol for the treatment of mild hypertension. *Lancet* **ii**, 539–43.

Medical Research Council Working Party (1985) M.R.C. trial of treatment of mild hypertension. Principal results. *BMJ* **2**, 97–105.

RAMSEY L. E. (1985) Mild hypertension: rest patients, not populations. *J. Hypertension* **3**, 449–457.

REID D. D., HAMILTON P. J. S., McCARTNEY P., ROSE G., JARRETT B. J. and KERN H, (1976) Smoking and other risk factors for coronary heart disease in British Civil Servants. *Lancet* **2**, 979–984.

ROSE G., *In:* The Hypertensive Patient. P 3. The Pitman Press, Bath. 1980.

ROUSE E. L. and BEILIN J., (1984) Vegetarian diet and blood pressure. *J. of Hypertension* **2**, 231–237.

VELASQUEZ M. T. AND HOFFMAN R. G., (1985) Overweight and Obesity in Hypertension. *Quart. J. Med.* **54**, 205–212.

Veterans administration co-operative study group in anti-hypertensive agents. (1967) *JAMA* **202**, 1028.

## Figures and Tables

**Figure f1:**
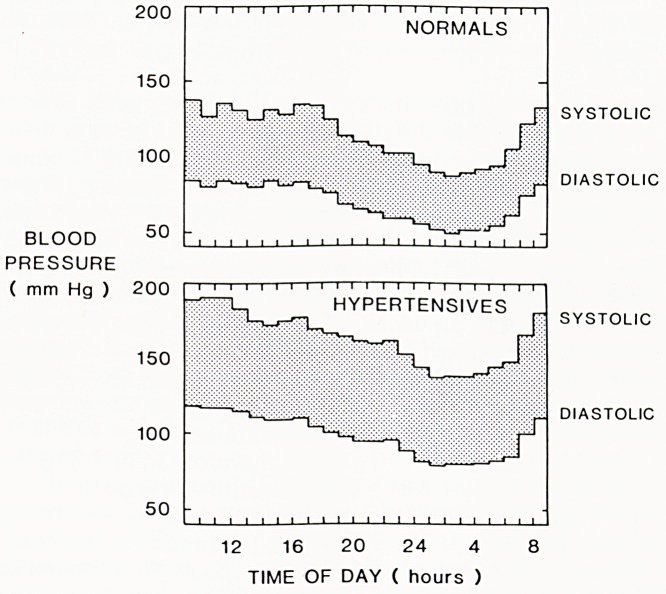


**Figure 2 f2:**
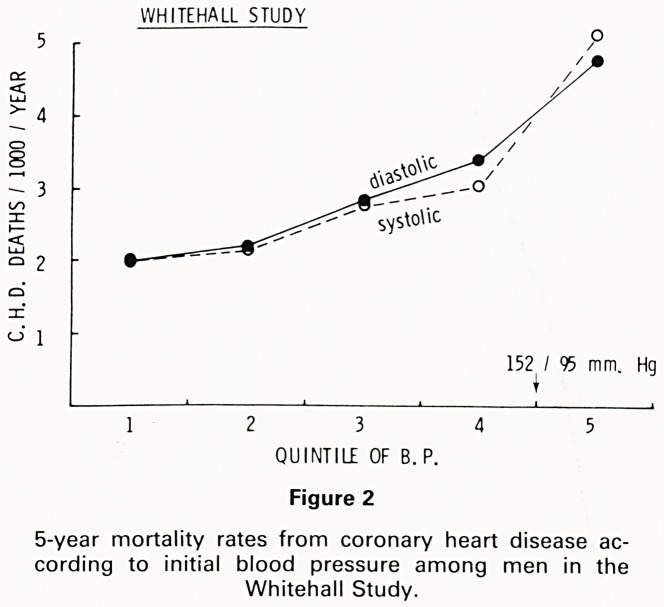


**Figure 3 f3:**
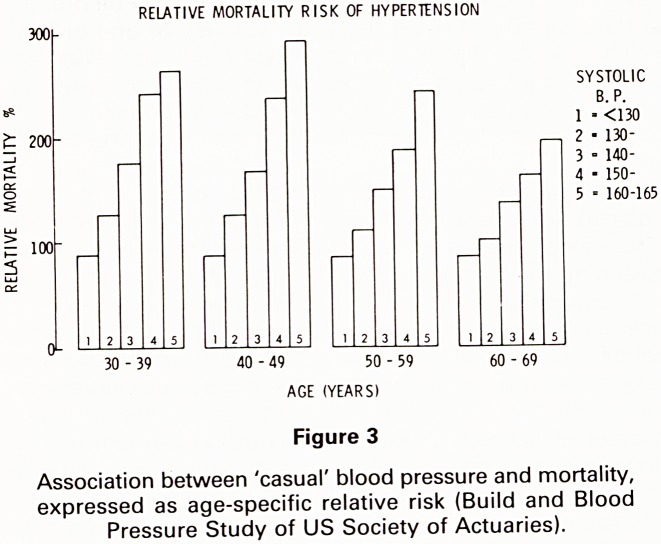


**Figure 4 f4:**
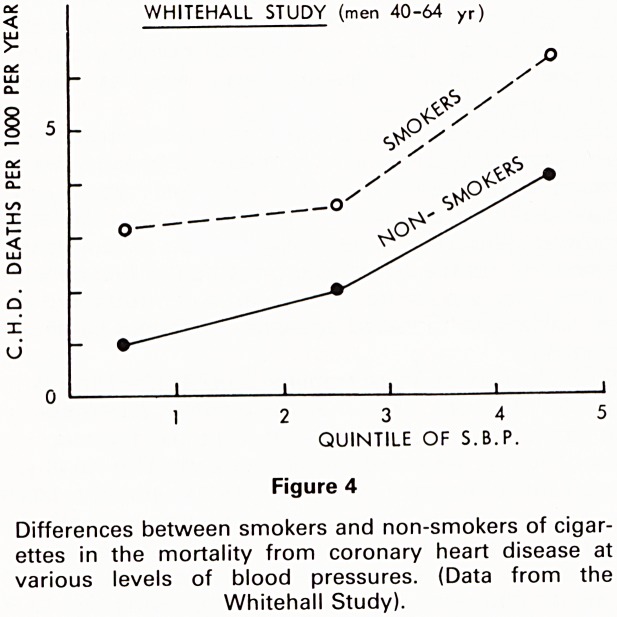


**Figure 5 f5:**